# Spin Trapping Analysis of Radical Intermediates on the Thermo-Oxidative Degradation of Polypropylene

**DOI:** 10.3390/polym15010200

**Published:** 2022-12-30

**Authors:** Thu Anh Nguyen, Hui Ming Lim, Kenji Kinashi, Wataru Sakai, Naoto Tsutsumi, Satoko Okubayashi, Satoru Hosoda, Tetsu Sato

**Affiliations:** 1 Doctor’s Program of Materials Chemistry, Graduate School of Science and Technology, Kyoto Institute of Technology, Matsugasaki Sakyo, Kyoto 606-8585, Japan; 2 Undergraduate School of Applied Chemistry, Kyoto Institute of Technology, Kyoto 606-8585, Japan; 3 Faculty of Materials Science and Engineering, Kyoto Institute of Technology, Kyoto 606-8585, Japan; 4 Faculty of Fiber Science and Engineering, Kyoto Institute of Technology, Kyoto 606-8585, Japan; 5 Graduate School of Science & Technology, Kyoto Institute of Technology, Kyoto 606-8585, Japan; 6 Tohoku Electronic Industrial Co., Ltd., Mukaiyama 2-14-1, Sendai, Miyagi 982-0841, Japan

**Keywords:** polypropylene (PP), thermo-oxidative degradation, electron spin resonance (ESR), spin trapping method, supercritical carbon dioxide (scCO_2_), radical intermediates, chemiluminescence

## Abstract

The purpose of this study is to investigate the thermo-oxidative degradation behavior of polypropylene (PP) by comparing three types of pristine PP granules (consisting of homopolymer, random copolymer, and block copolymer) with their corresponding oxidized analogues. These analogues were intensely oxidized under oxygen at 90 °C for 1000 h by using the electron spin resonance (ESR) spin trapping method that can detect short-lived radical intermediates during the degradation. The degrees of oxidation could be evaluated by chemiluminescence (CL) intensity, which was related to the concentration of hydroperoxide groups generated in the PP chain. In the pristine PP samples, a small amount of hydroperoxides were found to be formed unintentionally, and their homolysis produces alkoxy radicals, RO•, which then undergo *β*-scission to yield chain-end aldehydes or chain-end ketones. These oxidation products continue to take part in homolysis to produce their respective carbonyl and carbon radicals. On the other hand, in the oxidized PP granules, because of their much higher hydroperoxide concentration, the two-stage cage reaction and the bimolecular decomposition of hydroperoxides are energetically favorable. Carbonyl compounds are formed in both reactions, which are then homolyzed to form the carbonyl radical species, •C(O)–. PP homopolymer produced the largest amount of carbonyl radical spin adduct; thus, it was found that the homopolymer is most sensitive to oxygen attack, and the presence of ethylene units in copolymers enhances the oxidation resistance of PP copolymers.

## 1. Introduction

**Scheme 1:**
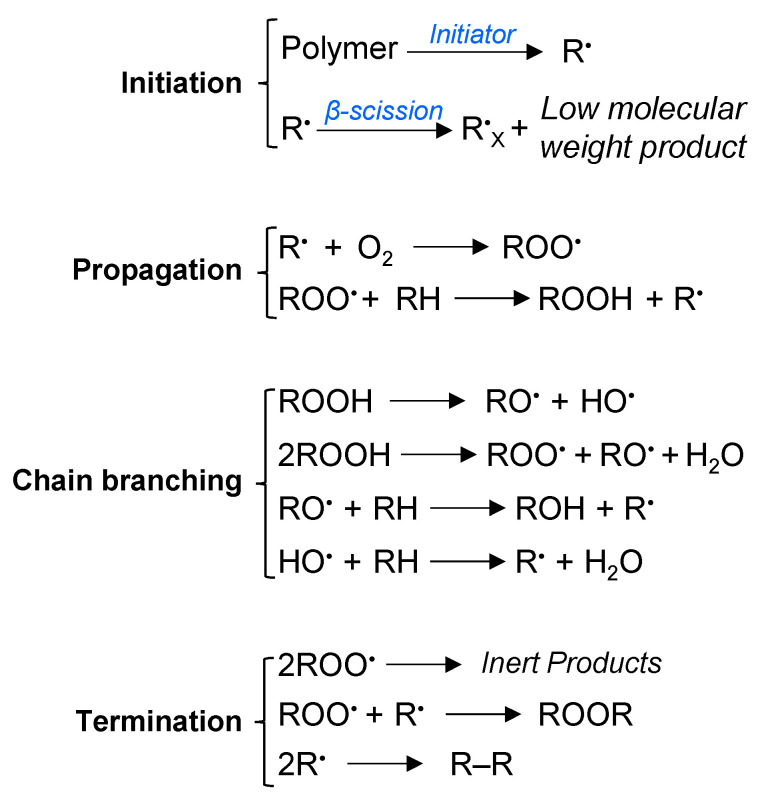
The basic autooxidation scheme (BAS) proposed by Bolland and Gee.

PP is well-known to be highly susceptible to air oxidation. To produce PP-based products after polymerization, fabrication methods are performed under air at elevated temperatures, such as extrusion, injection, blowing, spinning, or compression. These processes initiate and accelerate the oxidative degradation of molten PP and sometimes lead to an intense loss in mechanical properties. The basic autooxidation scheme (BAS) of polymers, first proposed by Bolland and Gee, was derived from their studies on rubber and lipids. As shown in Scheme 1 [1,2,3,4], oxidation progresses via reactions between oxygen and the carbon-centered radicals, producing peroxyl (ROO•). The production of new radicals via the decomposition of hydroperoxide (ROOH) plays a critical role, which leads to the autocatalytic behavior.

The existence of ROOH in PP has been detected through various methods. For example, Gijsman et al. [5] applied iodometric titration [6] to investigate the decomposition of peroxides in PP between 50 and 90 °C. They found that there are two stages in the autoxidation of PP in this low-temperature range: an induction period in which oxidation is controlled by slowly decomposing peroxides, and a fast oxidation period in which oxidation is governed by peracids. Qian et al. [7] investigated the thermal degradation of isotactic PP (i-PP) at 190 °C, utilizing gel permeation chromatography (GPC) and Fourier-transform infrared (FTIR). The relationship between the carbonyl index and the probability of broken chains reveals that kinetically favored scission occurs near the oxygen-centered radical moieties. Dauphin et al. used liquid phase carbon-13 nuclear magnetic resonance (^13^C-NMR) analysis to identify hydroperoxide, acids, alcohols, and ketone groups as the main products from the photo- and thermo-initiated oxidation of i-PP [8]. The dominant presence of methyl ketones and carboxylic acids among the carbonyl compounds was confirmed. However, even though numerous studies have been conducted to understand the thermo-oxidative degradation mechanism of PP, there is still debate about it because it is difficult to predict the detailed reaction pathways only using the results from product analysis.

In such a case, direct information about the intermediate radicals involved in the degradation process might be helpful in a deep investigation of the degradation mechanism and the development of anti-degradation measures. Electron spin resonance (ESR) spectroscopy, with its high sensitivity and specificity to radical compounds, has been predicted to be a suitable method for analyzing the intermediate radicals produced during the degradation of polymer materials [9,10,11,12,13]. However, the studies of polymer degradation using ESR have been limited by the short lifespan of intermediate radicals. To overcome this limitation, a specific method, namely spin trapping, can be used [11,14,15]. When a specific short-lived free radical is trapped by a spin-trapping reagent, a distinctive ESR spectrum of the spin adduct with a lifespan long enough for ESR measurements is obtained. The hyperfine structure (*hfs*) and hyperfine coupling constant (*hfcc*) of the ESR spectrum allow the molecular structure around the unpaired electron to be determined. In our laboratory, we have used the ESR spin trapping method to investigate the degradation of common polymer materials, such as poly(vinyl alcohol) (PVA) [16] and poly(butylene terephthalate) (PBT) [17], as well as the related copolymer elastomer poly(butylene terephthalate)-*co*-poly(ethylene oxide) (PBT-*co*-PEO) [18]. In a recent study, we have investigated the thermal degradation of PP fabrics under nitrogen between room temperature (RT) and 220 °C [19]. The spin trapping reagent was impregnated by a swelling method using supercritical carbon dioxide (scCO_2_) since PP is insoluble in organic solvents under mild conditions. It was revealed that the non-oxidative thermal degradation of PP starts at tertiary carbon positions via homolysis to form •CH_3_ and –CH_2_–•CH–CH_2_–, or via hydrogen abstraction to form –CH_2_–•C(CH)_3_–CH_2_–. These radicals are then involved in bond cleavage reactions, producing secondary carbon radicals. Interestingly, it was found that the PP fabric was unexpectedly oxidized, as evidenced by the detection of alkoxy radicals, possibly during the manufacturing process.

As described above, the thermo-oxidative degradation of PP materials has not been investigated via direct observation of radical species. Thus, in this study, we used the ESR spin trapping method to investigate the effect of oxidation on the thermal degradation mechanism of PP using antioxidant-free granules that were intensely oxidized by keeping them at 90 °C for 1000 h under an oxygen flow. Isotactic PP granules of three different types (homopolymer, random, and block copolymers containing small amounts of ethylene units) before and after oxidation were inspected to identify differences in degradation behavior in incorporated ethylene units. In addition, chemiluminescence (CL) measurements were also used to evaluate the degree of oxidation in each sample. PP oxidation is known to be accompanied by a low level of CL emission [20,21,22], and CL measurements have been carried out to examine the very early stages of oxidation of PP [21,22,23,24,25,26,27,28]. Lastly, the thermo-oxidative degradation mechanism of PP was elucidated based on the ESR results with consideration of degree of crystallinity, thermal properties, polymer structures, and CL intensity.

## 2. Materials and Methods

### 2.1. Materials

Three types of isotactic PP granules (homopolymer (PP-H), random copolymer (PP-R), and block copolymer (PP-B)) were obtained from Sumitomo Chemical Co., Ltd. (Tokyo, Japan), as listed in Table 1. These granules contained no additives, such as antioxidants or UV stabilizers. The spin trapping reagent TTBNB was purchased from Fujifilm Wako Pure Chemical Co. (Osaka, Japan), and 1,1,1,3,3,3-hexafluoro-2-isopropanol (HFIP) was purchased from Flourochem Ltd. (Glossop, UK); these chemicals were used as received. Deuterated chloroform (CDCl_3_) with tetramethylsilane (TMS) as an internal reference was purchased from Tokyo Chemical Industry Co., Ltd. (Tokyo, Japan).

### 2.2. Preparation of Oxidized PP

Five grams of each granule, PP-H, PP-R, or PP-B, were put in a Petri dish and placed in a stainless steel container connected to an oxygen flow maintained at a rate of 100 mL/min. The container was placed on a hot plate to heat the PP granules constantly at 90 °C for 1000 h. During this oxidation procedure, the PP granules were thoroughly mixed every 24 h to achieve uniform oxidation. After oxidation, the samples were kept in a dark vial for the subsequent experiments. The oxidized PP granules were designated as OPP-H, OPP-R, and OPP-B, as listed in Table 1.

### 2.3. Preparation of ESR Samples

The PP samples impregnated with TTBNB were prepared by a batch method with scCO_2_ treatment using high-pressure equipment (Model SCF-Sro, JASCO Co., Tokyo, Japan) as mentioned in a previous report [19]. The scCO_2_ method has also been used to introduce spin probes into polymer matrices to study the molecular motion and dispersion states in polymer solids from ESR spectral differences [29,30]. For each treatment, 200 mg of PP granules, 50 mg of TTBNB, and 0.25 mL (4.5 *v*/*v*%) of HFIP were placed into a cylindrical pressure vessel with a free volume of 4.5 cm^3^. The impregnation was carried out at 60 °C and 20 MPa for 2 h. The sample was then washed several times with ethanol to remove any TTBNB clinging to the granule surface before being vacuum dried for 3 hrs. TTBNB is sensitive to light; therefore, all the steps above were carried out in the dark.

### 2.4. Characterizations

#### 2.4.1. DSC Measurements

The thermal properties of the PP granules were characterized using DSC equipment (Q2000, TA Instruments, New Castle, DE, USA) over a temperature range of −30–200 °C at a heating rate of 5 °C/min under a nitrogen gas flow. The degrees of crystallinity (*χ*_c_) of the PP granules were determined from experimental heat of fusion (Δ*H*_m_), with an estimated heat of fusion for PP homopolymer value of 100% crystalline ($\Delta H_{m}^{0}$) as 185 J/g [31], using the following equation:

$$\chi_{c}=\frac{\Delta H_{m}}{\Delta H_{m}^{0}}\times 100$$

#### 2.4.2. FTIR Measurements

The chemical composition of PP was analyzed using FTIR spectroscopy (FTIR-4700, JASCO Co.) in attenuated total reflectance (ATR) mode. All the spectra were recorded in the wavenumber range 3500–500 cm^−1^ by averaging 128 scans with a resolution of 4 cm^−1^ at RT.

#### 2.4.3. TGA Measurements

The thermal weight loss of PP was characterized using TGA apparatus (Discovery TGA, TA Instruments). For TGA measurements, specimens of approximately 5 mg were placed on platinum TGA pans and heated from RT to 600 °C at a heating rate of 10 °C/min in both nitrogen and dry air.

#### 2.4.4. ^1^H-NMR Measurements

^1^H-NMR measurements were performed to determine the concentration of the impregnated spin trapping reagent, TTBNB. The details of this method have already been mentioned in a previous study [19]. The PP granules, after scCO_2_ treatment, were immersed in deuterated chloroform (CDCl_3_) for at least 18 h, and the extracted solution was measured by an NMR spectrometer (Avance 300, Bruker (Ettlingen, Germany). Measurements were performed at 300 K, with a 1 s relaxation delay, 30° pulse width, 2.648 s acquisition time, a scan number of 8, and a spectral width of 20.63 ppm. The concentration of TTBNB was estimated by collating the areas of its characteristic peaks with the calibration curve of TTBNB.

#### 2.4.5. ESR Measurements

A sample of 50 mg PP granules/TTBNB was prepared in a 5 mm diameter quartz tube with nitrogen purging before sealing. The radicals formed during the thermal degradation of PP were inspected using an ESR spectrometer (JES-TE300, JEOL (Tokyo, Japan)) with an X-band microwave at a microwave frequency of approximately 9.2 GHz and a microwave power of 2.0 mW. The modulation width was 0.1 mT at 100 kHz, and Mn^2+^/MgO was used as a magnetic field standard. The sweep time was four minutes, the time constant was 0.03 s, and the accumulation was one time. A thermal controller unit (DVT3, JEOL) precisely controlled the measurement temperature from RT to 250 °C. Each step of the temperature change took less than one minute. The ESR spectra were then analyzed using computer software (Excel, Microsoft) with basic theoretical Gaussian and Lorentzian functions. The obtained ESR spectra were reproduced by a computational approximation fitting routine, with manual adjustment of the peaks in the multiple ESR spectra.

#### 2.4.6. CL Measurements

CL emissions were observed with a single photon counting method using a Chemiluminescence Analyzer CL FS5 (Tohoku Electronic Industry Co., Ltd., Sendai, Japan). Samples of approximately 100 mg were placed on aluminum pans and put into the chamber of the CL equipment. The heating process was similar to that used for ESR measurements; isothermal measurements were taken under nitrogen at different temperatures from 60 to 240 °C with an increment of 20 °C. The CL intensity was normalized based on the sample weight.

## 3. Results and Discussion

### 3.1. Thermophysical Properties of PP

**Figure 1:**
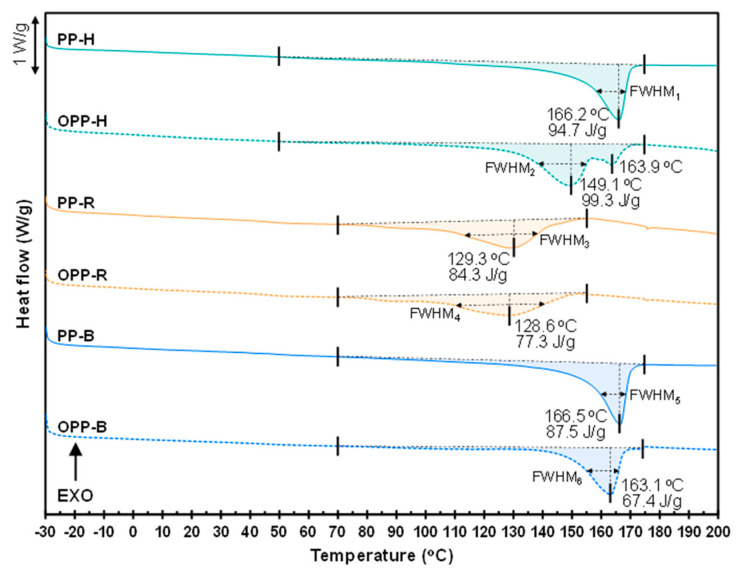
DSC thermograms of isotactic PP granules (homopolymer, random, and block copolymers) before and after oxidation at a heating rate of 5 °C under nitrogen.

The basic thermal properties of pristine and oxidized PP granules, namely the melting temperature, *T*_m_, and the heat of fusion, Δ*H*_m_, were identified by DSC measurements. The DSC curves obtained from the PP granules before and after oxidation are presented in Figure 1. A summary of the DSC results can be found in Table 2.

While PP-H and PP-B display approximately similar melting temperatures, the melting state of PP-R is at a much lower temperature of 129.3 °C, with a broader melting peak with a shoulder around 70–100 °C. This difference is due to the foreign ethylene units, which prevent the formation of the long helicoidal chain structures of the isotactic PP backbone necessary for a large crystalline structure. This effect is much less in PP-B due to the block structure of the copolymer. On the other hand, the effect of ethylene units on degree of crystallinity is limited, and the difference between the three PP samples was within 6%. The oxidized copolymer samples of OPP-R and OPP-B exhibit slightly lower melting temperatures compared to the correlative pristine granules. The reason for this result is thought to be that the hydroperoxide groups generated on the crystalline-to-amorphous boundaries converted the crystalline regions into an amorphous phase [28]. However, for OPP-H, another melting point, about 15 °C lower than the original peak, appeared. In OPP-H, chain reactions of decomposition in the propylene unit chain, which are not interfered with by ethylene units, are likely to have occurred. The peak with the lower melting temperature was assigned to the oxidized component, while the other was attributed to the not-yet-oxidized portion.

### 3.2. Chemical Analysis by FTIR Measurements

**Figure 2:**
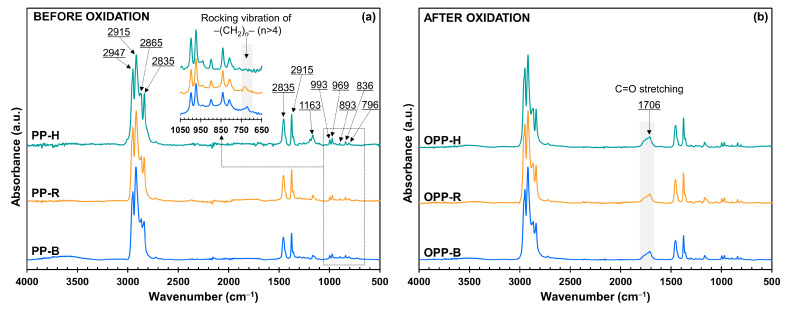
FTIR spectra of PP granules including homopolymer, random copolymer, and block copolymer (**a**) before and (**b**) after oxidation in the wavenumber range 4000–500 cm^−1^.

FTIR plots of PP granules before (a) and after oxidation (b) are presented in Figure 2, and the essential assignments are summarized in Table 3. As can be seen in Figure 2a, the FTIR spectra of the PP homopolymer, random copolymer, and block copolymer before oxidation contain similar prominent characteristic absorption peaks that are typical of PP materials. However, PP-R and PP-B show a small absorption peak at around 722 cm^−1^, while PP-H shows no such absorption. This peak can be attributed to the –(CH_2_)*_n_*– sequences with *n* > 4, which are equivalent to the sequences of more than two ethylene units and imply the existence of long-chain ethylene units in the structures of PP-R and PP-B [32].

After oxidation, the FTIR spectra of PP granules show evidence of a new absorption band in the range 1650–1790 cm^−1^, with a peak at 1706 cm^−1^, as shown in Figure 2b, confirming the presence of the C=O bonding in carbonyl compounds, from mostly ketones, in addition to aldehydes and esters, which demonstrates that oxidation occurred in the PP granules.

### 3.3. Thermal Decomposition Analysis by TGA

**Figure 3:**
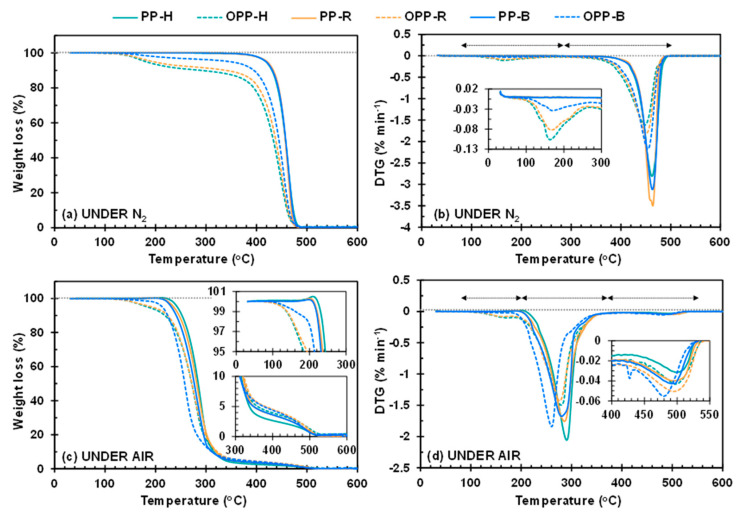
TGA and DTG curves for PP granules before (solid lines) and after oxidation (dashed lines) at a heating rate of 10 °C/min: (**a**) TGA and (**b**) DTG curves in nitrogen, (**c**) TGA and (**d**) DTG curves in air. The double arrows in (**b**,**d**) indicate the temperature ranges for the weight loss steps of the thermal decomposition of oxidized samples.

The thermal decomposition behavior of pristine granules PP-H, PP-R, and PP-B and oxidized granules OPP-H, OPP-R, and OPP-B was examined by TGA in both a nitrogen and an oxidative atmosphere of dry air flow. The TGA curves are depicted in Figure 3, with derivative thermogravimetric (DTG) curves calculated from the TGA data.

TGA curves of all pristine PP granules measured in nitrogen exhibit a similar single-step decomposition that starts at 280 °C and ends at 500 °C. On the other hand, the TGA curves measured in air show two stages of weight loss: one significant decrease that occurs from 220 °C to 360 °C and another slight decrease at temperatures above 360 °C. The significant decomposition stages in both environments can be attributed to random chain scissions taking place alongside the thermal degradation of polyolefins [38,39,40]. TGA curves in air also show an initial mass increase of 0.5% at around 200 °C due to oxygen uptake and the formation of hydroperoxides. These oxidized hydrocarbons are unstable at higher temperatures and rapidly turn into labile products. The mass increase is highest for PP-H, which shows that the homopolymer is more sensitive to oxygen attack than the other two PP copolymers. However, the thermal stability of PP-H from 200 °C to 300 °C is greater than that of PP-R and PP-B, which may be influenced by the difference in degree of crystallinity of each sample.

On the other hand, the TGA results for oxidized PP granules show a clear difference in the decomposition behavior between these samples. The decomposition curves in nitrogen can be roughly divided into two weight loss stages of 80–280 °C and 280–500 °C, but there were three weight loss stages of 80–200 °C, 200–370 °C, and 370–550 °C in air. In both environments, the weight loss during the first stage is approximately 10%, which can be attributed to the decomposition of hydroperoxides [41]. The thermal stability in the first stage is much greater for OPP-B than for OPP-R and OPP-H. The inclusion of ethylene and the block copolymer structure may account for the differences in stability. The greater weight loss of OPP-H again confirms that the PP homopolymer is more susceptible to oxygen attack than the copolymer samples. As mentioned in the DSC results, chain reactions in the PP chain are likely to have occurred. The second decrease shows the biggest drop for both environments, which is related to the cleavage reactions of the polymer chain. This major decomposition in air occurs at lower temperatures than in nitrogen. Interestingly, in air, the thermal stability of OPP-B suddenly decreases more rapidly than that of OPP-H and OPP-R at temperatures above 200 °C. This result indicates that the PE moiety in oxidized PP-B is less resistant to air above 220 °C. Usually, PE chains are considered to be more heat resistant than PP chains. To the best of our knowledge, no previous papers have described such a contrasting phenomenon. Finally, in air, one additional weight-loss stage of less than 10% occurred before complete decomposition at 550 °C; this stage may be attributable to oxidative breakdown of the residues [42].

### 3.4. Evaluation by ^1^H-NMR of the Effect of scCO_2_ Treatment

**Figure 4:**
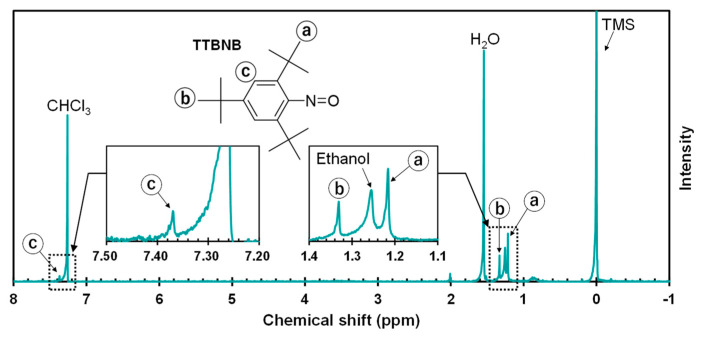
^1^H-NMR spectra of a CDCl_3_ extracted solution of PP granules (PP-H) after scCO_2_ treatment.

The concentration of TTBNB impregnated into PP granules by treatment with scCO_2_ was evaluated by ^1^H-NMR. As described in our previous study [19], the relative concentrations of TTBNB were estimated by comparing the integrals of the TTBNB characteristic signals at 1.21 ppm, as shown in Figure 4, to the concentration standard curves. The relative impregnated concentrations are presented in Table 4. The amount of uptake of TTBNB in PP-R is nearly double that of PP-H and PP-B. This is because the scCO_2_ treatment temperature of 60 °C was close to the melting temperature of PP-R (70–155 °C).

### 3.5. Analysis of Radicals by Spin Trapping ESR

#### 3.5.1. ESR Measurements

**Figure 5:**
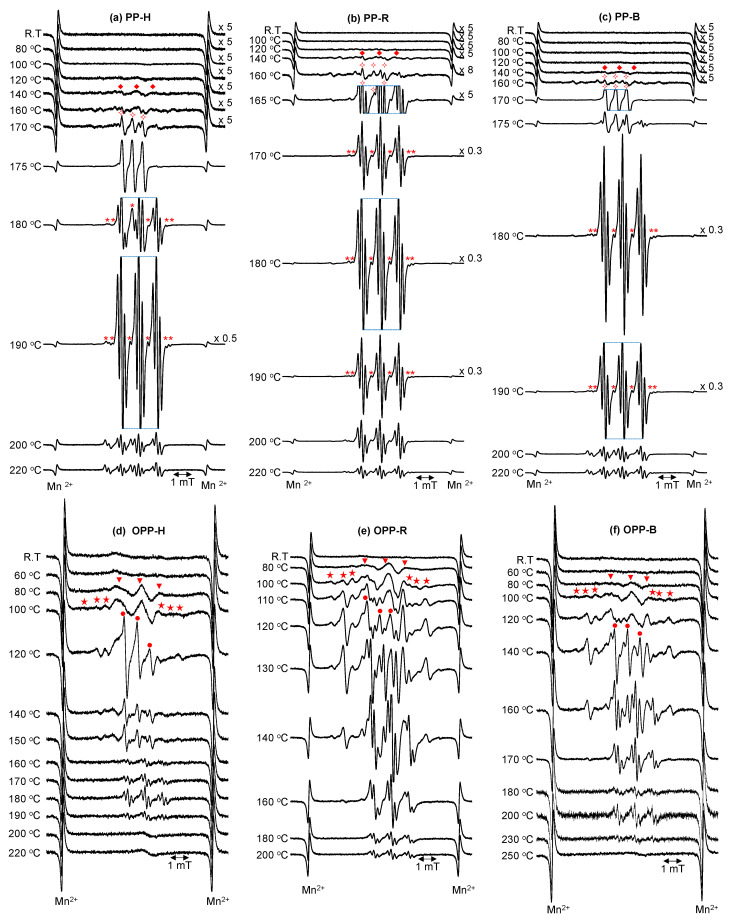
ESR spectra of pristine and oxidized PP granules impregnated with TTBNB during stepwise heating from RT to over 200 °C: (**a**) PP-H, (**b**) PP-R, (**c**) PP-B, (**d**) OPP-H, (**e**) OPP-R, and (**f**) OPP-B. The dashed lines represent spectra with the nine-line signal part-cut due to the limited accumulation range. The details of symbols, ◆, ✧, **＊**, ▼, ★, and ●, are described in the text.

The thermal dependence of the ESR spectra of pristine PP granules and oxidized PP granules impregnated with TTBNB by scCO_2_ treatment following stepwise heating under nitrogen from RT to over 200 °C is presented in Figure 5. Initially, almost no ESR signal is detected at RT. However, when the temperature was increased from RT to 200 °C or higher, ESR signals become visible for both pristine and oxidized samples, and the intensity and *hfs* with temperature change differently according to the samples. These changes in ESR spectra in Figure 5 demonstrate that TTBNB can capture the radical intermediates generated during the degradation of PP and turn them into spin adducts that are detectable by ESR measurements.

In these spectra, many signals show good symmetry, while some show a little asymmetry. This is caused by the slower molecular motion of the radical moiety of the spin adduct generated in the solid (or rubber state) PP matrix than in the liquid state [30]. However, because most of the peaks were analyzed by using simple equations as described below, it was not necessary to analyze the molecular motion state of the radicals.

All PP granules before oxidation display a similar temperature-dependent pattern, as can be seen in Figure 5a–c. Some small signals appear at 140 °C and continue to grow in intensity until they show symmetric *hfs* at 160 °C, peaking at 190 °C for PP-H and 180 °C for PP-R and PP-B. Afterwards, the peak intensities decay rapidly. With the same measurement conditions, the ESR spectra of the oxidized PP samples show a notable variation in the distinctive features of *hfs* compared to the pristine samples; the signals appear earlier and reach their highest intensity at a lower temperature compared to the pristine samples. A modest anisotropic signal, marked with down-pointing triangles, can be identified from 80 °C, and this signal increases in intensity at 100 °C for all the samples. At 100 °C, several additional signals, marked with a star symbol, develop on the outermost two sides of the anisotropic signals. The signals then change into symmetric shapes and reach maximum peak intensity at 120 °C for OPP-H, at 150 °C for OPP-R, and at 140 °C for OPP-B. Among the three oxidized PP granules, the ESR results show that OPP-R has the most complex response to temperature variations.

#### 3.5.2. Assignments of Spin Adducts

**Figure 6:**
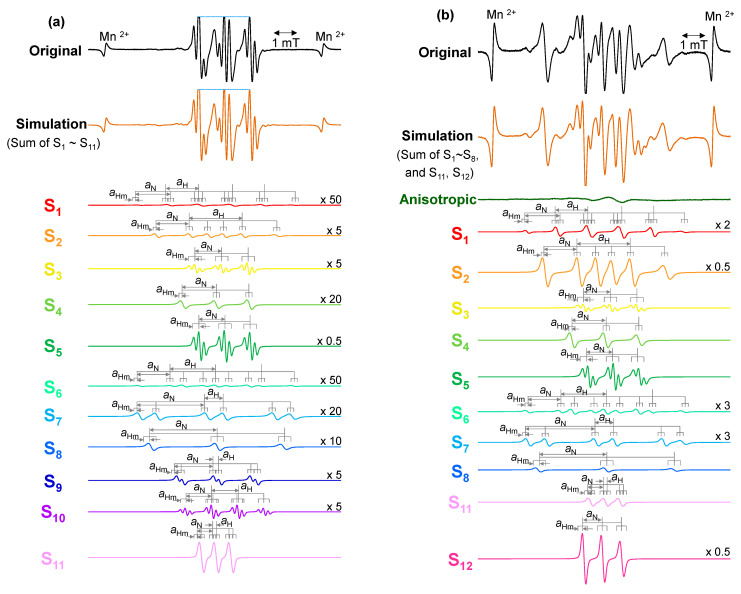
ESR spectra and simulated spectra of (**a**) pristine homopolymer PP-H observed at 180 °C, and (**b**) oxidized random copolymer OPP-R observed at 130 °C. The parameters *a*_H_, *a*_N_, and *a*_Hm_ are the hyperfine coupling constants (*hfcc)*.

To identify the molecular structures of the radical intermediates, each ESR spectrum in Figure 5 was deconvoluted into individual spin adduct components using the fundamental principles of hyperfine interactions between the unpaired electron and the surrounding magnetic nuclei. Figure 6a,b show representative simulation results for the ESR spectra of pristine and oxidized PP, respectively. Table 5 presents the spectrum characteristics for each spin adduct, including *hfcc*, *g*-values, and molecular structures, as well as the reference values. Most of the ESR spectra of pristine PP granules constitute some or all of the eleven spin adducts, S_1_–S_11_, in varying proportions. In the simulation for oxidized PP granules, the existence of an anisotropic component at low temperatures and a novel spin adduct, S_12_, was considered. The parameters of S_1_–S_10_ are almost similar to those detected in the thermal degradation of the PP fabric we have reported previously [19].

The spin adduct S_1_, which exhibits only six lines with *hfcc* (*a*_N_ = 1.3 mT, *a*_H_ = 1.2 mT, and *a*_Hm_ = 0.08 mT), was assigned to the •CH_3_ radical that was eliminated from the PP main chain. The spin adduct S_2_, which consists of 18 lines with two *hfcc* (*a*_N_ = 1.36 mT and *a*_H_ = 2.1 mT), was assigned to the secondary carbon radical in the chain, −CH_2_−•CH−CH_2_−, or at the chain end, •CH(CH_3_)–CH_2_–. The radical −CH_2_−•C(CH_3_)−CH_2_− was found to be represented by anilino-type spin adduct S_3_ (*a*_N_ = 1.05 mT, *a*_Hm_ = 0.193 mT, and *g*-value = 2.0046) and nitroxide-type spin adduct S_4_ (*a*_N_ = 1.33 mT, *a*_Hm_ = 0.08 mT, and *g*-value = 2.0059). The spin adduct S_5_, which was ascribed to an anilino-type spin adduct of a tertiary butyl carbon radical, •C(CH_3_)_3_, is independently produced by the thermal self-decomposition of TTBNB and is not involved in the degradation of PP. The spin adduct S_6_, made up of 27 lines (*a*_N_ = 1.30 mT, *a*_H_ = 1.8 mT, *a*_Hm_ = 0.08 mT, and *g*-value = 2.0058), is derived from the radical •CH_2_–CH(CH_3_)–, produced by the main chain scission of PP. The spin adduct S_7_ shows a large value of *a*_N_, at 2.7 mT, which is commonly associated with spin adducts derived from oxygen radical species [18,43,44]. Therefore, it is proposed that S_7_ is formed from the alkoxy radical −CH_2_−CH(O•)−CH_2_−. S_8_, the spin adduct attributed to −CH_2_−C(CH_3_)(O•)−CH_2_−, also has a large value of *a*_N_, at 2.65 mT. No oxygen atoms are in the original molecular structure of PP, and the FTIR spectra of the pristine PP samples in Figure 2a show no clear signal for an oxygen functional group. In our laboratory, we always keep our samples under light shielding and nitrogen flow. Therefore, the presence of the S_7_ and S_8_ components demonstrates that the PP main chain in pristine samples had already been attacked by oxidation to form oxygen radical species during processes before the sample arrived at our laboratory. For the spectra of pristine PP samples observed at around 170 °C or higher, the small signals indicated by the asterisk symbol in Figure 5a–c could be replicated by adding two new components, S_9_ and S_10_. The spin adduct S_9_ is identified as nitroxide-type by the *g*-value of 2.0057 derived from •CH(OH)– radical, whereas S_10_ is identified as anilino-type, derived from the same secondary carbon radical by a *g*-value of 2.0038.

In Figure 5a–c, for the pristine PP samples, a new spin adduct S_11_, indicated by the white concave-side diamond symbols, was not observed in our previous work. S_11_ consists of 18 lines with *a*_N_ = 0.59 mT, *a*_H_ = 0.09 mT, and *a*_Hm_ = 0.05 mT. However, S_11_ appears to show only three lines due to the small values of hydrogen splitting. This narrow value of *a*_N_ is close to the *hfcc* of the spin adduct from polyhalomethyl radicals •CX(O) reported by Hartgerink et al. [51]; thus, S_11_ is assigned to a formyl radical •CH(O) that can be produced from aldehyde products.

On the other hand, the ESR spectra for the oxidized PP samples in Figure 5d–f show additional triplet signals with *a*_N_ = 0.75 mT, indicated by the circle symbols, that can be detected easily around 120–150 °C. The formation of •C(O)C_6_H_11_ in the reaction system of cyclohexanol and lead tetraacetate using MNP resulted in an ESR spectrum with *a*_N_ = 0.78 mT, as reported by Kapustina et al. [52]. We propose that this new component, designated S_12_, is produced from the acyl radical •C(O)–, since its shape and *hfcc* parameters are similar to the reference value.

**Figure 7:**
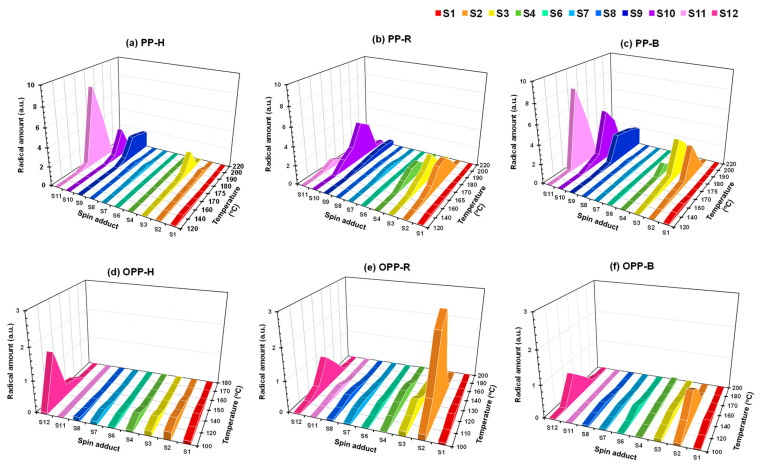
Radical amounts of spin adducts produced during stepwise heating of PP granules, arranged by temperatures for each spin adduct: (**a**) PP-H, (**b**) PP-R, (**c**) PP-B, (**d**) OPP-H, (**e**) OPP-R, and (**f**) OPP-B.

Based on the radical assignments, the radical amount of each spin adduct observed was determined by double spectra integration from the simulated ESR spectrum. The correlation of radical amounts between different PP samples was quantitatively evaluated by normalizing for the intensity of standard Mn^2+^ signals, the sample weight, and the impregnated concentration of TTBNB. Figure 7 shows the temperature dependence of radical amounts of spin adducts in a detailed 3D style. The amounts of S_5_ and the anisotropic component mentioned above are not included; this is because the former, S_5_, derived from the decomposition of TTBNB, is irrelevant to the degradation of PP, and for the latter, the precise calculation of the radical amount is difficult due to its asymmetric *hfs*.

It should be noted that the amounts of spin adduct radicals detected in the oxidized PP samples were lower than in the pristine PP samples. This is contrary to the expectation that more radical intermediates would be spin-trapped in the oxidized sample due to decomposition and subsequent radical chain reactions from the hydroperoxide groups. There are two possible reasons for this. One is that the recombination rate of oxygen radical species generated by peroxide decomposition, such as RO• and •OH, is much higher than the trapping rate, and the other is that nitroso-type spin trapping reagents, such as TTBNB, are generally suited to trapping carbon-centered radicals and not oxygen-centered radicals as is the case with nitrone-type spin trapping reagents, such as 5,5-dimethyl-1-pyrroline-*N*-oxide (DMPO). Regardless, the most characteristic feature of the oxidized PP samples is that S_2_ and S_12_ were observed around the melting temperature in particular, while S_2_–S_4_ and S_9_–S_11_ appeared in abundance in the pristine samples.

### 3.6. Chemiluminescence from PP Oxidation

**Figure 8:**
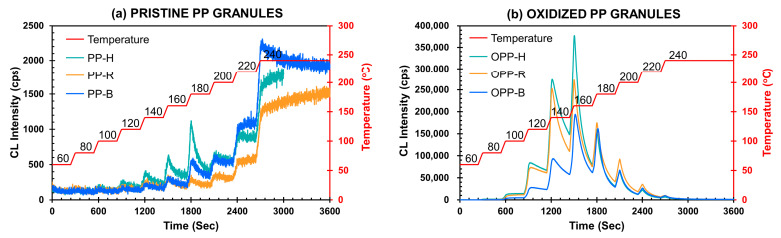
Chemiluminescence intensity of (**a**) pristine and (**b**) oxidized PP granules measured at increments of 20 °C for four minutes from 60 to 240 °C in nitrogen.

The stepwise heating CL curves in nitrogen for pristine and oxidized PP granules, together with temperature profiles from 60 to 240 °C, are shown in Figure 8. The pristine samples in Figure 8a show a tendency for the CL intensity to increase with increasing temperature, reaching approximately 2000 cps, which indicates that thermal oxidation occurred in pristine PP samples before measurement. The intensity temporarily reaches a maximum between 160 °C and 180 °C, and PP-H has a higher CL intensity than the other samples. These results confirm that oxidative degradation occurs to a limited extent in all the pristine samples and a little more in PP-H. On the other hand, when the oxidized PP granules were measured under the same conditions, the CL intensity was found to increase rapidly and reach a maximum at 160 °C, with ca. 400,000 cps for OPP-H, before decaying entirely at 240 °C, as shown in Figure 8b. This strong emission pattern implies that the degree of oxidation in oxidized samples is much more severe than in pristine samples, and homopolymer PP is most affected by oxygen, while random copolymer PP is oxidized more easily than block copolymer PP. Notably, the CL intensity behavior is correlated with the amounts of spin adducts derived from oxygen radical species S_7_, S_8_, and S_11_ in pristine samples and S_7_, S_8_, and S_12_, in oxidized samples. This correlation is discussed later in connection with the peroxidation reaction mechanism. Generally, the intensity of CL emission does not have a one-to-one correlation with the spin trap reaction, so quantitative analysis is not possible. However, similar trends can be qualitatively observed because the CL mechanism is related to oxidative degradation.

### 3.7. Reaction Mechanism of PP Thermo-Oxidative Degradation

Since the rate and yield of the spin trapping reaction are affected by the reactivity and diffusion of the original radical species, the observed amounts of radicals as spin adducts do not necessarily correspond to the original radical intermediates produced by degradation. However, carefully considering the circumstances of the spin adducts, the original formation and decay behavior of the trapped radicals can allow discussion of the degradation mechanism.

**Scheme 2:**
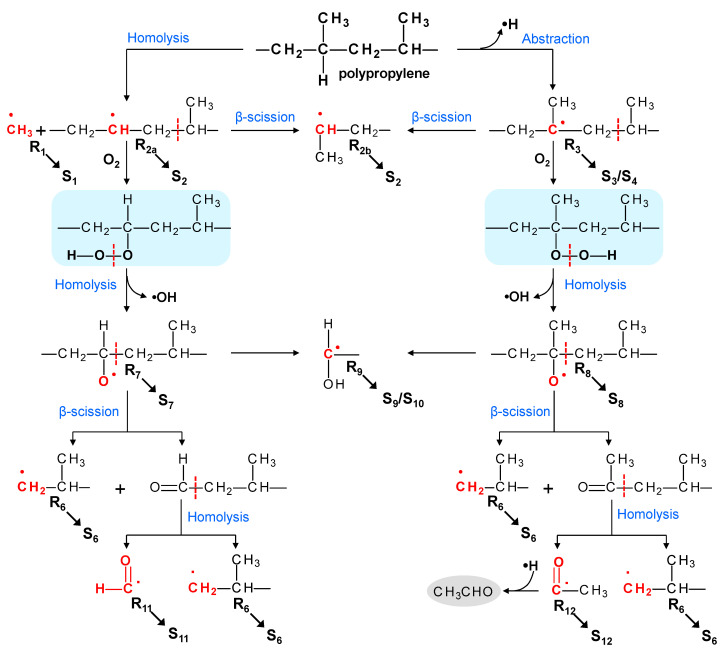
The inferred reaction scheme for the thermo-oxidative degradation of pristine PP.

The thermo-oxidative reaction mechanism of pristine PP derived in this study is summarized in Scheme 2. First, we conclude that the thermal degradation of pristine homopolymer follows the same path as previously reported PP fabric [19]. This is because the temperature dependence of the radical amounts of S_1_–S_10_ is the same in both cases.

The thermal decomposition begins at the tertiary carbon positions in two directions at around 100−120 °C; homolysis produces the methyl radical, •CH_3_ (R_1_), along with –CH_2_−•CH−CH_2_− (R_2a_), and hydrogen abstraction produces −CH_2_−•C(CH_3_)−CH_2_− (R_3_). The intermediate radical species R_1_ and R_2_ correspond to spin adducts S_1_ and S_2_, respectively, while radical R_3_ gives rise to two distinct spin adducts, S_3_, an anilino-type, and S_4_, a nitroxide type. S_1_ was most intense at around 140 °C before decaying completely at 180 °C. The radicals R_2a_ and R_3_ may then be subjected to the *β*-scission reaction, resulting in the formation of the secondary carbon radical •CH(CH_3_)− (R_2b_), also known as spin adduct S_2_, which appeared to a considerably greater extent in the copolymers PP-R and PP-B than in the homopolymer PP-H. This may be due to the high content of ethylene units in the copolymers being attacked by other radicals via hydrogen abstraction. Further degradation of PP is unlikely in the absence of oxygen. However, if oxygen is available, the carbon radicals R_2a_ and R_3_ react with it to form hydroperoxides, –ROOH. The homolysis of hydroperoxide begins at around 100 °C, producing the alkoxy radicals RO• (R_7_ and R_8_) and •OH. These alkoxy radicals successively undergo *β*-scission, yielding chain-end aldehydes and ketones and the primary alkyl radical •CH_2_–CH(CH_3_)– (R_6_). The two new spin adducts, S_11_ and S_12_, observed in this study may come from the further homolysis of those chain-end carbonyl compounds, producing the formyl radical •CH(O) (R_11_) and the acyl radical •C(O)CH_3_ (R_12_), respectively. The dominance of homolysis from the chain-end aldehyde and chain-end ketone is supported by differences in bond dissociation energies; HC(O)–CH_3_ is lower than H–C(O)CH_3_ and CH_3_C(O)–C_2_H_5_ is lower than CH_3_–C(O)CH_3_ [53]. The presence of S_11_ from •CH(O) was detected in considerable amounts in pristine PP samples but only in minor amounts in oxidized PP samples; this contrast can be explained by considering that a significant amount of peroxides produced a large number of active oxygen radicals, which are easier to deactivate by recombination than by trapping by TTBNB. On the other hand, S_12_ from •C(O)– was observed only in the oxidized samples. This is because the ketone groups were originally produced much more in the oxidized sample, not only at the chain end but also in the middle of the main chain, as will be discussed below. When the PP chain reaches a flexible state close to melting, •CH(OH)– (R_9_) is produced and detected by ESR as spin adducts **S_9_** and **S_10_**. As we suggested above, R_9_ is thought to be formed through hydrogen transfer and *β*-scission from R_7_ or R_8_, RO• [19]. The radical amounts of S_9_ and S_10_ detected in this study were higher than in PP fabric, which might be attributed to the higher sensitivity of PP granule samples to oxidation than PP fibers.

**Scheme 3:**
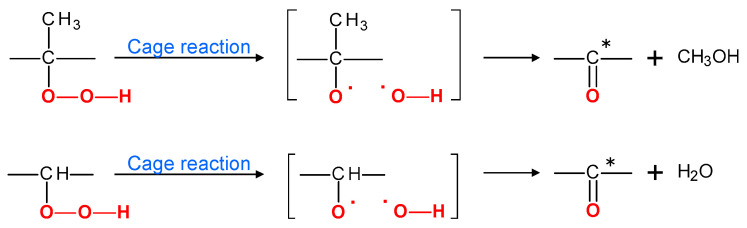
The cage reactions of hydroperoxides. Asterisks * indicate excited states.

**Scheme 4:**
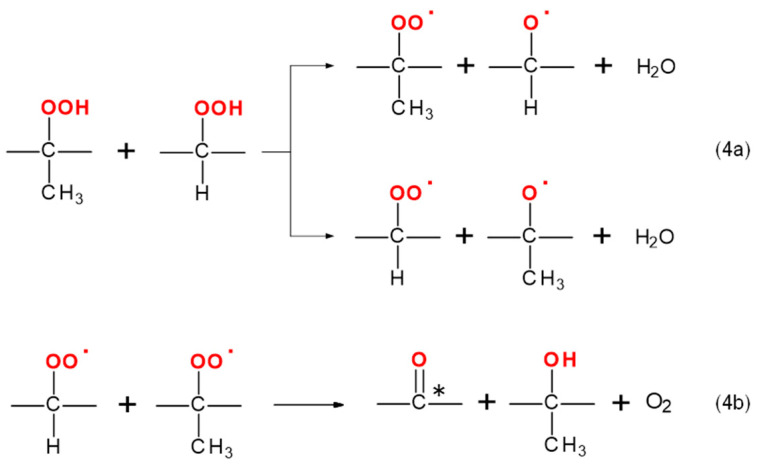
The bimolecular decomposition of hydroperoxides. The asterisk * indicates an excited state.

**Scheme 5:**
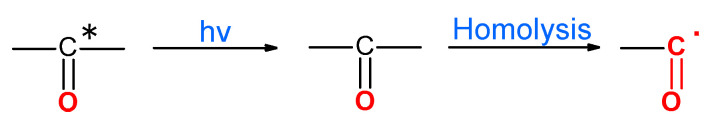
The formation of a carbonyl radical from the excited or ground state of a carbonyl group. The asterisk * indicates an excited state.

The FTIR, CL, and ESR analyses of oxidized PP granules demonstrate that the degree of oxidation in each oxidized sample is much higher than in the corresponding pristine sample. The differences observed in ESR spectra suggest that oxidation of OPP-H, OPP-R, and OPP-B follows quite a different route from the mechanism described in Scheme 2. Considering the significant signals of carbonyl groups detected by FTIR, extensive oxidation was likely to take place before measurements by the special reactions shown in Scheme 3 and Scheme 4. In Scheme 3, carbonyl groups result from a two-stage process of radical cage entrapment, with disproportionation of the products from homolysis of the peroxide O–O bond [54]. Furthermore, as illustrated in Scheme 4, the bimolecular decomposition of hydroperoxides is energetically favorable at excessive hydroperoxide concentrations, producing peroxide radicals ROO•, alkoxy radicals RO•, and H_2_O. The subsequent bimolecular reaction of ROO• results in the formation of carbonyl groups [55]. The carbonyl radical, •C(O)– (R_12_), is produced by homolysis adjacent to the carbonyl group at around 120–140 °C, as shown in Scheme 5, and is detectable by ESR.

**Scheme 6:**
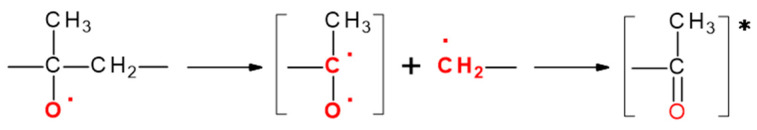
Formation of excited carbonyl group via a transient biradical state. The asterisk * indicates an excited state.

One reason to explain the high CL intensity observed in the oxidized PP samples should be that many hydroperoxide groups are present in addition to carbonyl groups. While the formation of excited state carbonyl groups from hydroperoxides shown in Scheme 3 and Scheme 4 plays an important role, another mechanism for CL is also possible as shown in Scheme 6; that is, the *β*-scission of RO• via the formation of a transient biradical state produces a carbonyl group in an excited state [55].

Among the oxidized PP samples, the largest amount of S_2_ from −•CH− was found in OPP-R. In the molecular structure of a random copolymer, the ethylene units are irregularly inserted between the propylene units along the polymer chain, whereas the ethylene units in a block copolymer tend to form phase-separated microstructures of ethylene propylene rubber (EPR) and polyethylene (PE) in the PP region [4,56]. The decomposition of these hydroperoxides yields the low molecular weight radical •OH, which is necessary for the additional hydrogen abstraction to neighboring carbon atoms. This process may be easier in random copolymers than in block copolymers, where the block ethylene chain forms a crystalline domain.

As seen in Figure 5d–f, anisotropic components with asymmetric *hfs* can be observed in the oxidized PP samples from 80 °C to 100 °C. The amount of this component cannot be evaluated by simulation because it is extremely difficult to analyze the asymmetric *hfs*, in contrast to spin adducts S_1_–S_12_. However, a rough calculation based on the spectrum at 80 °C shows that the amount of the anisotropic component is about 5~7 (a.u.) based on the same scale in Figure 7, and is nonexistent at temperatures over 130 °C. On the other hand, the radical amounts of S_2_ from −•CH− and S_12_ from •C(O)− shown in Figure 7 rapidly increase at 120 °C. Thus, the anisotropic components are probably the polymeric S_2_ and S_12_ components when their molecular motion is suppressed.

## 4. Conclusions

We applied the spin trapping method to investigate the short-lived radical intermediates that were produced during the thermo-oxidative degradation of PP. The samples were three types of pristine PP granules, including homopolymers and random and block copolymers, and their corresponding oxidized granules, which were oxidized by placing them under an oxygen flow at 90 °C for 1000 h. As a result, we found that alkoxy radicals, aldehyde radicals, carbonyl radicals, and various other carbon radicals derived from peroxidation were produced in different amounts depending on the degree of peroxidation. No other paper has directly analyzed the radical species produced in the oxidative degradation of polymer materials.

The degradation in pristine PP starts at the tertiary carbon atoms and proceeds in two ways: hydrogen abstraction, producing –CH_2_−•CH−CH_2_−, or homolysis, producing −CH_2_−•C(CH_3_)−CH_2_−. The main pathway for PP degradation is *β*-scission from these radicals. However, a small amount of oxidation was found unintentionally even in pristine PP granules, leading to the formation of the respective hydroperoxides, the presence of which was confirmed by CL measurements. Due to the very low concentration of hydroperoxide in pristine samples, oxidative degradation occurs through homolysis of the hydroperoxides, producing the alkoxy radicals –CH_2_–CH(O•)–CH_2_– and –CH_2_–C(CH_3_)(O•)–CH_2_– that participate in a subsequent *β*-scission reaction to produce chain-end aldehydes or ketones alongside •CH_2_–CH(CH_3_)–. The •CH(O) radical was detected as a result of chain-end aldehyde homolysis.

In oxidized PP granules, because the concentration of hydroperoxide is very high, degradation by a two-stage cage reaction and bimolecular decomposition of hydroperoxides are more favorable. The high possibility that these reactions occur is supported by the observation of CL intensities that were more than a hundred times stronger than those in the pristine PP. The radical •C(O)–R, detected by the spin trapping method, is derived from the carbonyl compounds formed by those reactions mentioned above and is regarded as the key radical species in oxidized PP degradation.

The ESR spectra also show how ethylene units in the copolymer structures affect the thermal degradation of PP; copolymers with ethylene units exhibit a higher amount of –CH_2_−•CH−CH_2_− and less •C(O)–. PP homopolymer produced the largest amount of carbonyl radical spin adduct; therefore, it was found that the homopolymer is the most sensitive to oxygen attack, and the presence of ethylene units in copolymers enhances the oxidation resistance of PP copolymers.

## Figures and Tables

**Table 1:** Polypropylene granule samples used in this study.

Type	Sample Code	Polymer Structure	Oxidation Status
Homopolymer	PP-H	No ethylene units	None
	OPP-H		Oxidized by oxygen flow at 90 °C
Random copolymer	PP-R	Contained ~5 wt% of ethylene units	None
	OPP-R		Oxidized by oxygen flow at 90 °C
Block copolymer	PP-B	Contained ~14 wt% of ethylene units	None
	OPP-B		Oxidized by oxygen flow at 90 °C

**Table 2:** Thermal properties of PP granules (homopolymer, random, and block copolymers) before and after oxidation: *T*_m_, Δ*H*_m_, *χ*_c_.

Sample	*T*_m_ (°C)	Δ*H*_m_ (J/g)	χ_c_ (%)	FWHM (°C)	Remarks
PP-H	166.2	94.7	51.2	9.0 ± 0.1	No ethylene units
OPP-H	149.1	99.3	53.7	16.7 ± 0.1	
	163.9				
PP-R	129.3	84.3	45.6	24.8 ± 0.1	5 wt% of ethylene units
OPP-R	128.6	77.3	41.8	30.3 ± 0.1	
PP-B	166.5	87.5	47.3	8.8 ± 0.1	14 wt% of ethylene units
OPP-B	163.1	67.4	36.4	11.9 ± 0.1	

**Table 3:** FTIR characteristic peak wavenumbers of PP before and after oxidation.

Abs. Band (cm^−1^)	Vibration Type	Assignment	Refs.
2947/2865	Asym./sym. stretching	CH_3_	[33,34]
2915/2835	Asym./sym. stretching	CH_2_	[33,34]
1452/1347	Asym./sym. bending	CH_3_	[33,34,35]
1163	Wagging/rocking	C–H/CH_3_	[33,34]
993/969	Rocking/stretching	CH_3_/C–C	[33,34,35]
893/796	Stretching	C–C	[33]
836	Rocking	C–H	[33,34]
722	Rocking	–(CH_2_)*_n_*–	[32,36]
1650–1790	Stretching	C=O	[33,37]

Abs.: absorption, asym.: asymmetric, sym.: symmetric.

**Table 4:** Correlated TTBNB concentration determined by ^1^H-NMR characteristic peak integrals.

Sample	Peak Integral (a.u.)	TTBNB Concentration (%)
	at 1.21 ppm	
PP-H	12.13	0.60
OPP-H	12.89	0.63
PP-R	23.68	1.18
OPP-R	23.24	1.21
PP-B	13.02	0.65
OPP-B	13.23	0.68

**Table 5:** Spectral parameters of the spin adducts **S_1_**–**S_12_** for PP/TTBNB heated from RT to 250 °C, and the literature data used to assign them.

Spin Adduct	Trapped Radical	*hfcc* (mT)			*g*-Value	Investigated Materials in Reference	Ref
		*a*_H_	*a*_N_	*a*_Hm_			
**S**_1_	•CH_3_	1.20	1.30	0.08	2.0059		
	•CH_3_ (ref)	1.23	1.30	0.081	2.0060	di-*tert*-butyl-peroxide/ TTBNB/benzene	[45]
**S**_2_	•CH(CH_3_)− or −CH_2_−•CH−CH_2_−	2.10	1.36	0.08	2.0058		
	−CH_2_−•CH−CH_2_− (ref)	2.16	1.33	N/A	2.0061	paraffin *n*-C_12_C_26_/TTBNB	[43]
**S**_3_	−CH_2_−•C(CH_3_)−CH_2_−	-	1.05	0.20	2.0046		
	−CH_2_−•C(CH_3_)−CH_2_− (ref)	-	1.05	0.193	2.0046	*n*-eicosane/TTBNB	[46]
**S**_4_	−CH_2_−•C(CH_3_)−CH_2_−	-	1.33	0.08	2.0059		
	−CH_2_−•C(CH_2_−)−CH_2_− (ref)	-	1.37	N/A	2.0060	LDPE/nitrosodurene/ benzophenone	[47]
**S**_5_	•C(CH_3_)_3_	-	1.02	0.20	2.0038		
	−CH_2_−•C(CH_2_−)−CH_2_− (ref)	-	1.03	0.19	2.0039	paraffin *n*-C_24_C_50_/TTBNB	[43]
**S**_6_	•CH_2_−CH(CH_3_)−	1.80	1.30	0.08	2.0058		
	•CH_2_−CH_3_ (ref)	1.799	1.346	0.083	2.0060	(*n*-Bu)_3_Sn-Br(or I)/ TTBNB/benzene	[45]
**S**_7_	−CH_2_−CH(O•)−CH_2_−	0.75	2.70	0.080	2.0059		
	•O−CH(CH_3_)_2_ (ref)	0.183	2.50	0.085	2.0057	CH_3_COOCH(CH_3_)_2_/TTBNB	[48]
**S**_8_	−CH_2_−C(CH_3_)(O•)−CH_2_−	-	2.65	0.08	2.0057		
	•O−C(CH_3_)_3_ (ref)	-	2.68	N/A	N/A	DBPO/nitroso-*t*- butane/benzene	[49]
**S**_9_	•CH(OH)−	0.19	1.49	0.06	2.0057		
	•CH(OH)CH_3_ (ref)	0.2	1.488	N/A	N/A	lactic acid, dibenzoyl peroxide/DMSO+H_2_O/MNP	[50]
**S**_10_	•CH(OH)−	1.06	1.03	0.18	2.0038		
	N/A	N/A	N/A	N/A	N/A	N/A	
**S**_11_	•CH(O)	0.09	0.59	0.05	2.0059		
	•CH(O) (ref)	0.14	0.69	N/A	2.0072	CHI_3_/2-nitroso-2- methylpropane (MNP)	[51]
**S**_12_	•C(O)–	-	0.75	0.06	2.0064		
	•C(O)(CH_2_)_4_CH_3_ (ref)	-	0.78	N/A	N/A	cyclohexanol/Lead tetra- acetate (LTA)/MNP	[52]

## Data Availability

Not applicable.
